# Persistent Müllerian duct syndrome: a rare clinical image

**DOI:** 10.11604/pamj.2023.44.45.38847

**Published:** 2023-01-24

**Authors:** Yarraiahgari Maheswara, Revat Meshram

**Affiliations:** 1 Department of Pediatrics, Jawaharlal Nehru Medical College, Datta Meghe Institute of Health and Educational Research, Sawangi Meghe, Wardha, Maharashtra State, India,; 2 Department of Paediatrics, Datta Meghe Institute of Higher Education and Research, Sawangi, Wardha, Maharashtra, India

**Keywords:** Müllerian duct, undescended testis, fertility

## Image in medicine

Persistent Müllerian duct syndrome (PMDS) is a rare form of internal male pseudo-hermaphroditism in which Müllerian duct derivatives are seen in a male patient. The syndrome is caused by an insufficient amount of Müllerian inhibiting substance (MIS) or due to insensitivity of the target organ to MIS. It is usually discovered during surgery for hernia or undescended testis, as *hernia uteri inguinalis*. Persistent Müllerian duct syndrome is often familial, presenting as an autosomal recessive trait. It was characterized by the presence of Müllerian structures (fallopian bed by Nilson in 1939, as *hernia uteri inguinalis*. It has fallopian tubes, uterus and upper part of vagina) in phenotypically and genotypically male (46XY). The gene responsible is chromosome 19. A 17-year-old male child presented with complaints of swelling in the right groin and associated with pain. On examination, an irreducible right inguinoscrotal swelling and right testis not palpable. Intraoperatively, the hernial sac was found to contain testis and uterus along with fallopian tubes and ovaries. The right testis was smaller compared to the opposite side. Excision of the Müllerian duct derived structures was done. This was followed by orchidopexy of right testis and right inguinal hernioplasty. Postoperative recovery of the patient was uneventful. It presents as inguinal hernia or undescended testis. A high index of suspicion is required when a patient presents with bilateral inguinoscrotal problems. Whenever possible, excision of Müllerian structures meticulously, avoiding injury to the vas, must be performed. The aim is to preserve fertility. Orchidopexy should be the goal of management.

**Figure 1:**
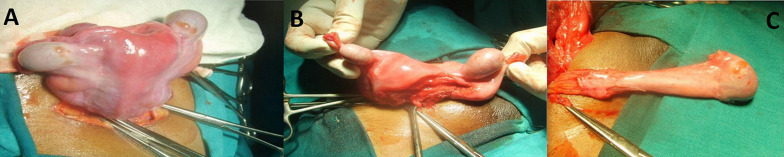
A) uterus with bilateral testis; B) surgical separation of uterus from testis; C) testis separated from uterus

